# New insights into muscle activity associated with phantom hand movements in transhumeral amputees

**DOI:** 10.3389/fnhum.2024.1443833

**Published:** 2024-08-30

**Authors:** Manon Chateaux, Olivier Rossel, Fabien Vérité, Caroline Nicol, Amélie Touillet, Jean Paysant, Nathanaël Jarrassé, Jozina B. De Graaf

**Affiliations:** ^1^ISM, Aix Marseille University, CNRS, Marseille, France; ^2^IRR, UGECAM Nord- Est, Nancy, France; ^3^U1150 Agathe-ISIR, CNRS, UMR 7222, ISIR/INSERM, Sorbonne University, Paris, France

**Keywords:** amputation, phantom movements, residual muscle activity, neural reorganization, rehabilitation

## Abstract

**Introduction:**

Muscle activity patterns in the residual arm are systematically present during phantom hand movements (PHM) in transhumeral amputees. However, their characteristics have not been directly investigated yet, leaving their neurophysiological origin poorly understood. This study pioneers a neurophysiological perspective in examining PHM-related muscle activity patterns by characterizing and comparing them with those in the arm, forearm, and hand muscles of control participants executing intact hand movements (IHM) of similar types.

**Methods:**

To enable rigorous comparison, we developed meta-variables independent of electrode placement, quantifying the phasic profile of recorded surface EMG signals and the specificity of their patterns across electrode sites and movement types.

**Results:**

Similar to the forearm and hand muscles during IHM, each signal recorded from the residual upper arm during PHM displays a phasic profile, synchronized with the onset and offset of each movement repetition. Furthermore, the PHM-related patterns of phasic muscle activity are specific not only to the type of movement but also to the electrode site, even within the same upper arm muscle, while these muscles exhibit homogeneous activities in intact arms.

**Discussion:**

Our results suggest the existence of peripheral reorganization, eventually leading to the emergence of independently controlled muscular sub-volumes. This reorganization potentially occurs through the sprouting of severed axons and the recapture of muscle fibers in the residual limb. Further research is imperative to comprehend this mechanism and its relationship with PHM, holding significant implications for the rehabilitation process and myoelectric prosthesis control.

## 1 Introduction

Phantom mobility has been described as the ability to move the phantom limb after amputation. It has been reported by 76% of upper limb amputees and persists over many years after surgery (Touillet et al., 2018). Despite common clinical assumptions, there is no inherent link between phantom mobility and phantom pain. Moreover, engaging in phantom movement training can even effectively alleviate phantom pain (Brunelli et al., 2015; Ortiz-Catalan et al., 2013, 2014). In a recent review, Scaliti et al. (2020) conceptualized phantom movements as “real movements of a dematerialized [limb].” Indeed, contrary to motor imagery, phantom mobility is systematically associated with muscle contractions in the residual limb (Raffin et al., 2012), even visually apparent on the surface of the residual limb. Furthermore, similarly to what is known about intact movements, when an ischaemic block is applied on the residual limb to suppress somatosensory feedback, the sensation of movement disappears while muscle contractions can remain (Reilly et al., 2006). Finally, muscle activity patterns in the residual limb are specific to the type of phantom movement performed (Reilly et al., 2006) and can be classified (Jarrassé et al., 2018), even for individual finger movements in transhumeral amputees (Jarrassé et al., 2017a,b). Thus, phantom mobility is a robust phenomenon, and, yet the underlying neurophysiological mechanisms are still poorly understood.

Previous studies on phantom mobility did not provide insights into the neurophysiological origin of the associated muscle activities on the residual upper arm during PHM. In some studies, muscle activities were recorded with a single pair of electrodes on one head of the residual biceps and triceps muscles (Gagné et al., 2009; Reilly et al., 2006). Despite this limited spatial resolution, they showed that muscle activity patterns varied across hand, wrist, and elbow phantom movements, but the origin of these activities could not be investigated. The more recent studies that used a much higher spatial resolution allowing to classify EMG patterns for up to 8 different types of phantom hand movements (Jarrassé et al., 2017a,b) demonstrated that signals from pairs of electrodes placed *on the same upper arm muscle* could provide relevant information about de type of executed phantom movement. Yet, the underlying neurophysiological origin is poorly understood given the ambiguity surrounding information on which classifiers base their classification. Therefore, by adopting a neurophysiological perspective in the analysis of muscle activity patterns associated with phantom mobility in transhumeral amputees, the aim of this study was to characterize their nature.

It was proposed that muscle activities in the residual upper limb during PHM could be new emerging activity due to reorganization of the nervous system at different levels after amputation (Gagné et al., 2011). Yet, it is also possible that such muscle contractions only reflect persisting synergistic activity originally associated with contractions of the -now missing- limb muscles. For example, synergistic activity are reported in the deltoid and in bi-articular biceps and triceps muscle groups for stabilizing the upper limb at the shoulder and elbow joints during intact hand movements (IHM) (McKiernan et al., 1998). If such synergistic activity indeed persists after amputation, muscle activity in the residual upper arm during PHM should be similar to the one observed during IHM in the intact upper arm, which would give information about the origin of these muscle activities. Therefore, to better characterize the nature of the muscle activity patterns associated with PHM, we compared those occurring during PHM with those occurring during IHM. We chose not to analyze IHM of the amputees’ contralateral intact arm because the compensatory use of the residual limb in daily life might influence intact arm muscle control (Garbarini et al., 2018; Makin et al., 2013). Instead, we used a control group of non-amputated participants and verified with one amputee that muscle activities in the intact upper arm during IHM were similar to those recorded in the control participants.

## 2 Materials and methods

### 2.1 Participants

Six participants (one female, five males) with a traumatic unilateral transhumeral amputation (P1–P6, 24–75 years old) and seven controls (C1–C7, aged 24–55 years) were included in the study. To be included, amputees had to be able to mimic their PHM with their intact hand in real time. Amputees who experienced phantom limb pain while performing PHM were excluded as this has been shown to limit their ability to move the phantom limb (Gagné et al., 2009). The delay since amputation varied from 2 to 48 years. Five of the six amputees used a myoelectric prosthesis daily. Their control relies on the generation of muscle activity in the residual triceps and/or biceps, allowing the EMG signal recorded by the two electrodes placed inside the prosthetic cuff to detect when a certain threshold is reached. The contraction of one muscle is needed to initiate a movement in one direction or the other, while the co-contraction of both muscles is required to switch the mode from hand closure/opening to wrist rotation, and vice versa. P5 was the only one to own a poly-digital prosthesis but its control was similarly sequential: modifications of finger movement configurations were obtained by successive co-contractions of the two muscles. Demographic data of the amputees are summarized in Table 1.

**Table 1:** Demographic data concerning the six amputated participants.

	Sex	Age (years)	Delay since amputation (years)	Amputated side	Pain treatment	Myoelectric prosthesis	Mobilization capacity
P1	F	75	14	Right	No	Yes	F1, F2, F345, Hand, Pinch
P2	M	42	5	Right	Yes	Yes	F1, Hand, Pinch
P3	M	73	48	Left	No	Yes	Hand
P4	M	49	3	Left	Yes	No	F1, F345, F2, Hand
P5	M	24	2	Left	Yes	Yes	F1, F5, Pinch, Hand
P6	M	32	4	Right	Yes	Yes	F5, Hand, F2, F1, F3, Pinch

M, male; F, female; Fn, finger flexion/extension with n going from 1 (thumb) to 5 (little finger); F345, simultaneous flexion/extension of F3, F4, and F5; Pinch, thumb/index opposition opening/closure; Hand, whole hand opening/closure. The different types of PHM are indicated in the order of execution during the recording session. This order was defined according to the self-reported difficulty of movement execution, starting with the easiest.

**Figure 1:**
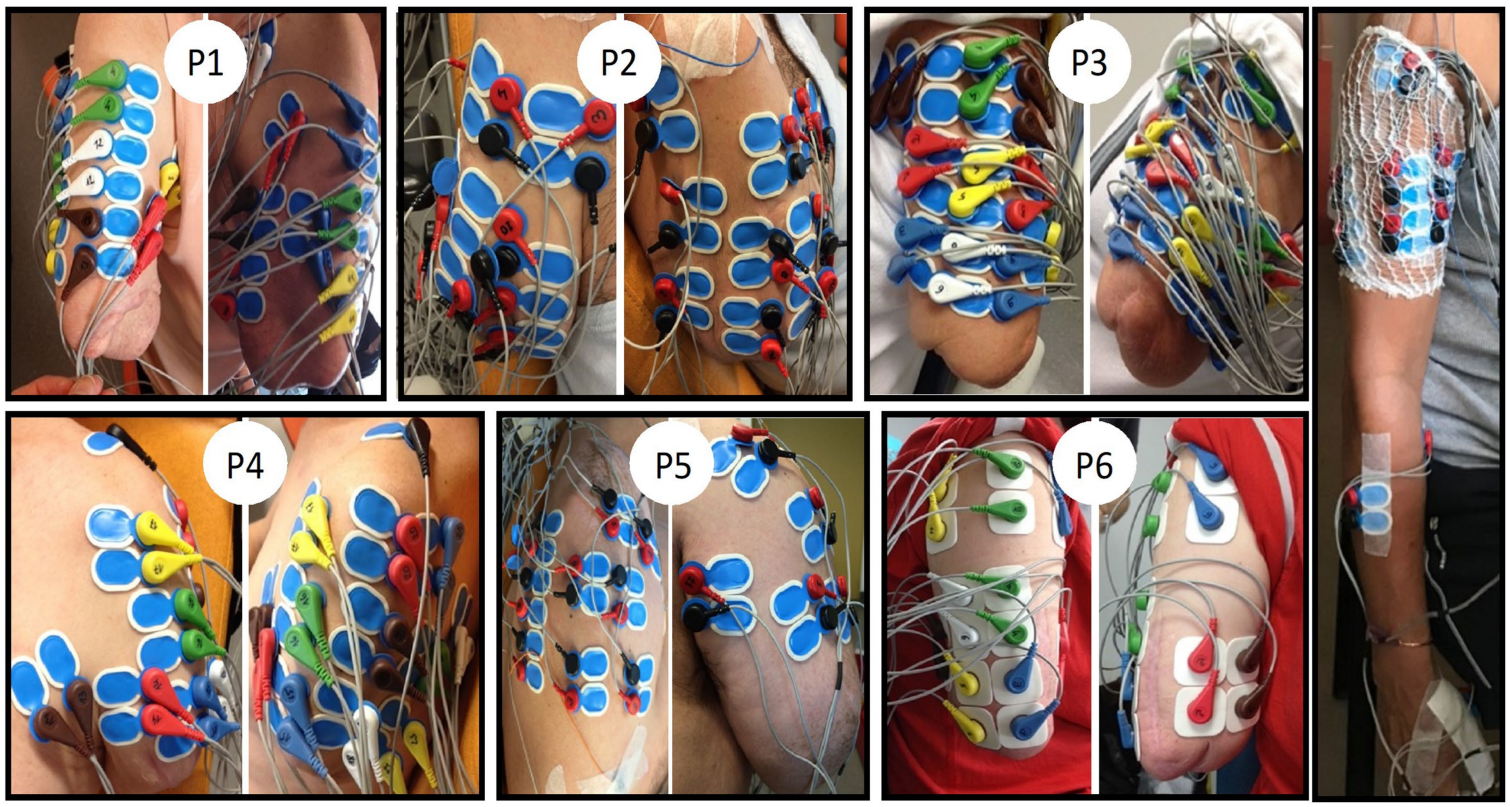
EMG electrode placements on the residual upper arm for the six amputees (PI to P6) and one control participant (right side). Only the electrodes placed on the biceps and triceps were selected for further analyses. Note the non-conventional placement of four pairs of electrodes on the biceps muscle of the control participant. The same was done on the triceps (not visible here). Participants provided their informed consent to the publication of these images.

All amputees were recruited at IRR (Nancy, France). The control participants were recruited from the research institute (Marseille, France). The experiments were carried out in accordance with the Declaration of Helsinki of the World medical association. Written informed consent was obtained from all participants and the study received approval from a Research Ethics Committee (CERES No. 2016-57). Participants also provided informed consent for the publication of photos of their body parts in Figure 1 while maintaining their anonymity.

### 2.2 Protocol

To determine the self-evaluated difficulty in executing each type of PHM, the recording session began with a short questioning based on a detailed semi-directed interview that had been conducted previously for each participant. Amputees were instructed to execute a series of 10 repetitions of a complete cycle of phantom movement (i.e., flexion/extension or closing/opening) at a self-chosen comfortable speed. They were also instructed to simultaneously mimic, symmetrically, and as accurately as possible, the movement amplitude and velocity with their intact contralateral hand equipped with a Cyberglove® II^1^ that recorded the kinematic data. This technique is widely used in the literature (De Graaf et al., 2016; Gagné et al., 2009; Reilly et al., 2006; Touillet et al., 2018). Movement execution began after the experimenter provided instructions on the type of movement to perform. Due to tremor or blocking [reported in the literature as an “impossibility to move despite strong effort” (De Graaf et al., 2016)] related to fatigue, for some phantom movements, the participants had to take breaks and temporally perform a different type of phantom movement before returning to the original one in order to complete the required number of 10 repetitions. Table 1 summarizes the different types of PHM each participant executed. We paid careful attention to preserve participants from fatigue by adjusting the duration of resting periods between series of movements to their needs. In addition, each recording session was videotaped for more accurate offline visual monitoring of tremor during phantom movement execution. If this occurred, the corresponding cycle of movement was rejected from analysis. The whole recording session lasted for about 90 min and took place in the presence of a medical doctor.

The control group performed series of 10 repetitions of the following cyclic movements with their dominant hand: flexion/extension of each of three individual fingers (thumb, index, and middle finger), and closing/opening of hand and pinch. In an attempt to closely match these IHM with the PHM of the amputated participants, the protocol included three distinctive features. First, the IHM were executed while keeping the upper arm and forearm in a relaxed vertical position (aligned with the trunk) to match the relaxed position of the residual upper arm of the amputees. Second, control participants were asked to perform movements at a slow speed to match the average speed of the PHM performed by the amputee group (6 s per cycle). This was trained beforehand, and the experimenters visually verified that the speed was maintained along the session. Finally, to rule out any potential influence of intact hand mimicking on the results of the comparison between controls and amputees, the control group was also asked to accurately mimic the amplitude and speed of the IHM with their contralateral hand in real time. Kinematics was also recorded via the Cyberglove® II the mimicking contralateral hand was equipped with. Thus, measurement procedures were identical for both groups: EMG activity was recorded on one upper arm while kinematics was measured on the mimicking contralateral hand.

### 2.3 Recordings

Pairs of surface EMG electrodes were placed at various sites on the residual limb, with the placement individualized for each amputee *based on the detection of muscle contractions through palpation during various types of PHM*. Electrodes were placed at each location where palpation could detect a contraction for at least one type of movement, within the constraints of available electrodes. Figure 1 shows photos with different views of the electrode locations. The present analysis focused on surface EMG signals recorded from 8 to 15 pairs of electrodes (depending on the length of the residual limb) placed distal to the deltoid muscles, i.e., only on the biceps and triceps brachialis muscles. For one amputee participant (P6), eight pairs of electrodes were also placed on similar locations on the biceps and triceps brachialis muscles of the contralateral intact arm.

For the control group, a total of 11 pairs of EMG electrodes were used for the analyses. It was not possible to precisely match the location of the electrodes on the right arm of controls to the one on the residual arm of amputees, primarily because the amputation has changed the anatomy of the upper arm muscles. Yet, we chose recording sites closely resembling those on the residual muscles of amputees (see the right side of Figure 1). Thus, for each control participant, four pairs of electrodes were placed on the biceps brachialis (BB) and four on the triceps brachialis (TB) of the right arm. For each of these muscle groups, one pair of electrodes was placed following the SENIAM recommendations (Hermens et al., 2000), a second pair was placed distal to the first one. Finally, the two other pairs were placed laterally to the previous two pairs but still on the same muscle. The three remaining pairs of electrodes were placed, respectively, on the superficial extensor and flexor muscles of the fingers (on the forearm) and on one intrinsic hand muscle (opponens pollicis, OP) following the SENIAM recommendations.

An ANT-Neuro® eego-sports system with shielded cables was used to record EMG activity at a sample frequency of 1 kHz continuously throughout the experimental session. A Cyberglove® II recorded the angular positions of the five fingers of the contralateral hand during real-time mimicking at a sample frequency of 100 Hz. Details about the calibration of the Cyberglove®II have been reported in the literature (De Graaf et al., 2016; Jarrassé et al., 2017b). Kinematic data and EMG data could be synchronized thanks to a dedicated push button which sent a marker to both recording systems at the start of each sequence of movement cycles.

Upon request, the raw data supporting the conclusions of this article will be made available by the authors, without undue reservation.

### 2.4 Analyses

Before going into details, two methodological issues must be mentioned. First, we did not assess PHM kinematics as such (i.e., amplitude, velocity), which has already been investigated in the literature (De Graaf et al., 2016). PHM kinematics were recorded only for the purpose of analyzing muscle activity patterns of each individual type of half-cycle (i.e., type of movement). Since in intact hand movements, agonist and antagonist muscle activities evidently differ in opposite movements (i.e., flexion versus extension of individual fingers, opening versus closing of pinch and hand), PHM needed to be analyzed in this way as well. Secondly, as the precise placement of EMG electrodes could not be standardized across participants (both within and between groups), we devised meaningful meta-variables that were independent of the exact electrode locations. This independence was essential for enabling group comparisons, allowing data averaging across participants within each group before comparing the two groups.

#### 2.4.1 Pre-processing

**Figure 2:**
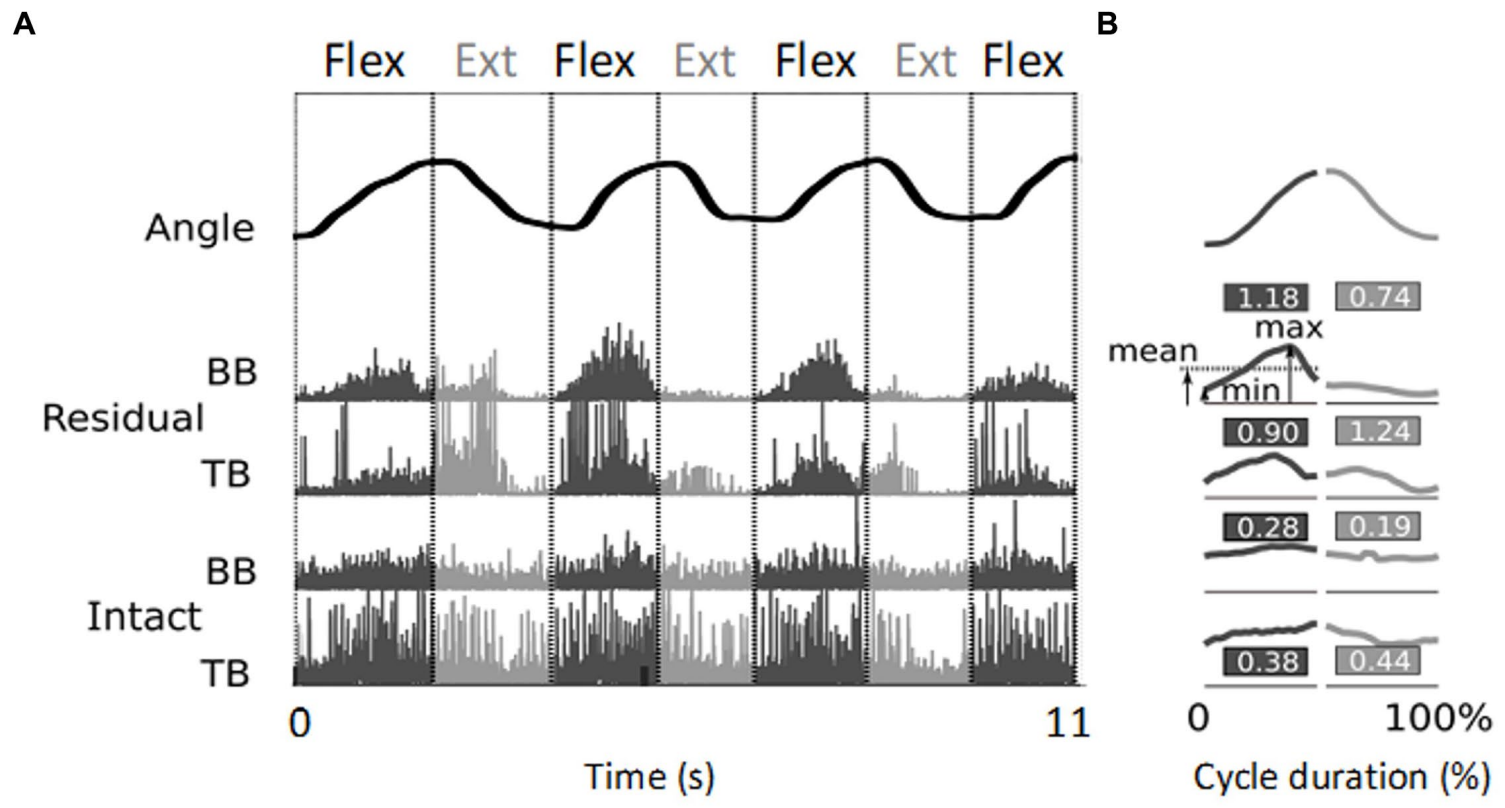
Illustration of EMG signal processing resulting in relative phasic muscle activity values. **(A)** The top trace shows the angular evolution of 3.5 cycles of flexion/extension of the little finger executed over 11 s. The four lower traces represent the corresponding rectified EMG activity normalized, for each electrode site, by the maximum value, recorded from the biceps (BB) and triceps (TB) brachialis muscles of the residual arm and intact upper arm, respectively. **(B)** Mean kinematic traces and EMG envelopes for each half cycle. The duration of each movement half-cycle is normalized to 50% of the total cycle time. The values indicate the relative phasic muscle activity calculated from the signals shown in panel **(A)** (see text). Note the higher values found for the residual upper arm compared to the intact upper arm.

Data collected with the Cyberglove® II was filtered with a 4th order Butterworth low-pass filter (cut-off frequency 2.5 Hz) and resampled at 1 kHz to match the EMG signal (see the upper trace in Figure 2A). The EMG signal was filtered using a 4th order Butterworth band-pass filter (10–400 Hz) and series of cyclic movements were separated. EMG amplitude envelopes were calculated via the root mean square of the EMG signal over a sliding time window of 0.5 s with 0.01 increments (Jarrassé et al., 2018, 2017b). Next, for each series of cyclic movements, we extracted the timing of phase changes from the kinematics, which were then utilized to cut the EMG activity into half cycles. Given the highly variable duration of each half cycle across different types of PHM and among participants, each half cycle repetition was normalized to a time-base representing 50% of the entire corresponding cycle. This step was necessary to later average the EMG envelopes over all repetitions for each given type of half cycle, which we will be referred to as “movement type” (e.g., thumb flexion, thumb extension, whole hand opening). The EMG envelopes are shown in Figure 2B. All kinematic and EMG analyses were done with the help of a custom-made MATLAB (version 2018b) script.

#### 2.4.2 Relative phasic muscle activity

Figure 2A shows typical examples of rectified EMG recordings. These recordings were obtained from two sites, one on the residual and one on the intact upper arm of participant P6 during flexion/extension of the phantom and intact little finger, respectively. During the PHM, clear phasic muscle activity was recorded from the biceps and triceps brachialis of the residual upper arm, whereas during the IHM, the intact upper arm showed mainly tonic muscle activity. Since the required task was to perform cyclic hand and finger movements and not to maintain postures, we developed a method to better distinguish phasic from tonic EMG activity. This was also used to normalize the amplitude of the recorded EMG signals instead of using the classical normalization to the maximal voluntary contraction, which was not applicable. Thus, in this cyclic action, the relative phasic muscle activity was quantified as follows: for each half cycle and recording site, the difference between the maximum and minimum values of the averaged envelope was divided by its mean value $\left(\frac{EMG_{\text{max}}-\;EMG_{\text{min}}}{\mathrm{mean}\left(EMG\right)}\right)$ . For each participant, each pair of electrodes and each type of phantom or intact hand movement, the mean relative phasic activity over the 10 repetitions was quantified. The obtained value was higher in case of purely phasic activity and lower when the bursts were superimposed on a tonic background (see Figure 2B for an illustration). For each participant, this value was averaged across all movement types and electrode sites to make inter- and intra-group comparisons of the relative phasic muscle activity between (i) the residual and intact upper arm, (ii) the residual upper arm and intact forearm, (iii) the intact upper arm and forearm.

#### 2.4.3 Movement-type and electrode-site specificities

**Figure 3:**
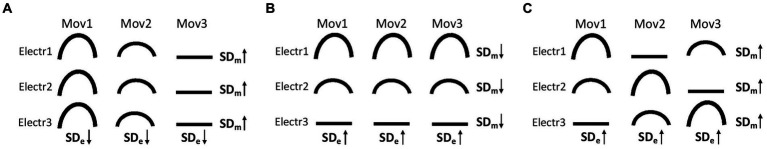
Three examples of schematic movement type (SD_m_), electrode site (SD_e_) and global (SD_E_ and SD_M_) specificities (**A–C**). ↑ and ↓ reflect, respectively, “high “and “low” specificities. **(A)** Low SD_E_ and high SD_M_, **(B)** high SD_E_ and low SD_M_, **(C)** high SD_M_ and high SD_E_.

In order to study the specificities of activity patterns in the residual upper arm muscles during PHM and compare these to muscle activity patterns in the intact upper arm during IHM, we developed two meta-variables: movement-type specificity and electrode-site specificity. First, the variation in relative phasic muscle activity across different movement types for a given pair of electrodes indicates whether this site is specifically activated for certain types of movements. We will refer to this as “*movement-type specificity*” and quantify it for each electrode site as the standard deviation of the relative phasic muscle activity values across different movement types (SD_m_). A high SD_m_ value indicates that this site is movement specific (see Figures 3A,C at each electrode site). Conversely, a low SD_m_ value indicates that the relative phasic muscle activity is similar across different types of movements at that site, indicating a lack of movement specificity (see Figure 3B at each electrode site). Secondly, the variation in relative phasic muscle activity across different electrode sites for a given type of movement reflects the specific involvement of certain muscle volumes located under these electrodes for this type of movement. We will refer to this as “*electrode-site specificity*” and quantify it for each type of movement as the standard deviation of the relative phasic muscle activity values across electrode sites (SD_e_, low in Figure 3A vs. high in Figures 3B,C). For each participant and arm segment (either forearm or upper arm), the “*global movement-type specificity*” (SD_M_) was calculated by averaging the SD_m_ values across all electrode sites. For a given participant, a high SD_M_ value indicates a high movement-type specificity at many electrode sites (as in Figures 3A,C). Yet, this quantification does not exclude that all electrodes may record the same relative phasic activity for a given type of movement (as in Figure 3A). Therefore, we also determined the “*global electrode-site specificity*” (SD_E_) by averaging the SD_e_ values over all movement types. To perform inter-group comparisons, values of *global movement-type* and *electrode-site specificities* (i.e., SD_M_ and SD_E_) of all participants were grouped and compared between PHM and IHM.

#### 2.4.4 Statistics

Because of the non-normal distribution of data, probably due to the restricted sample size related to the restricted population of transhumeral amputees, two-tailed comparisons between PHM and IHM were statistically tested using the non-parametric Wilcoxon Rank Sum test (independent samples, *n* = 6 for the amputees; *n* = 7 for the control group). The Wilcoxon Signed Rank test was used for the two-tailed *paired* comparison of relative phasic activity between the forearm and upper arm in control participants (*n* = 7). Statistical testing was performed in R-4.3.2 with the significance threshold set at 0.05, except for the two comparisons concerning the Relative phasic activity of the residual arm where it was rectified at 0.025. The values of test statistics, the *p*-values and the confidence intervals of the estimator (i.e., the pseudo-median, see Hollander et al., 2014) were all given by R.

## 3 Results

**Figure 4:**
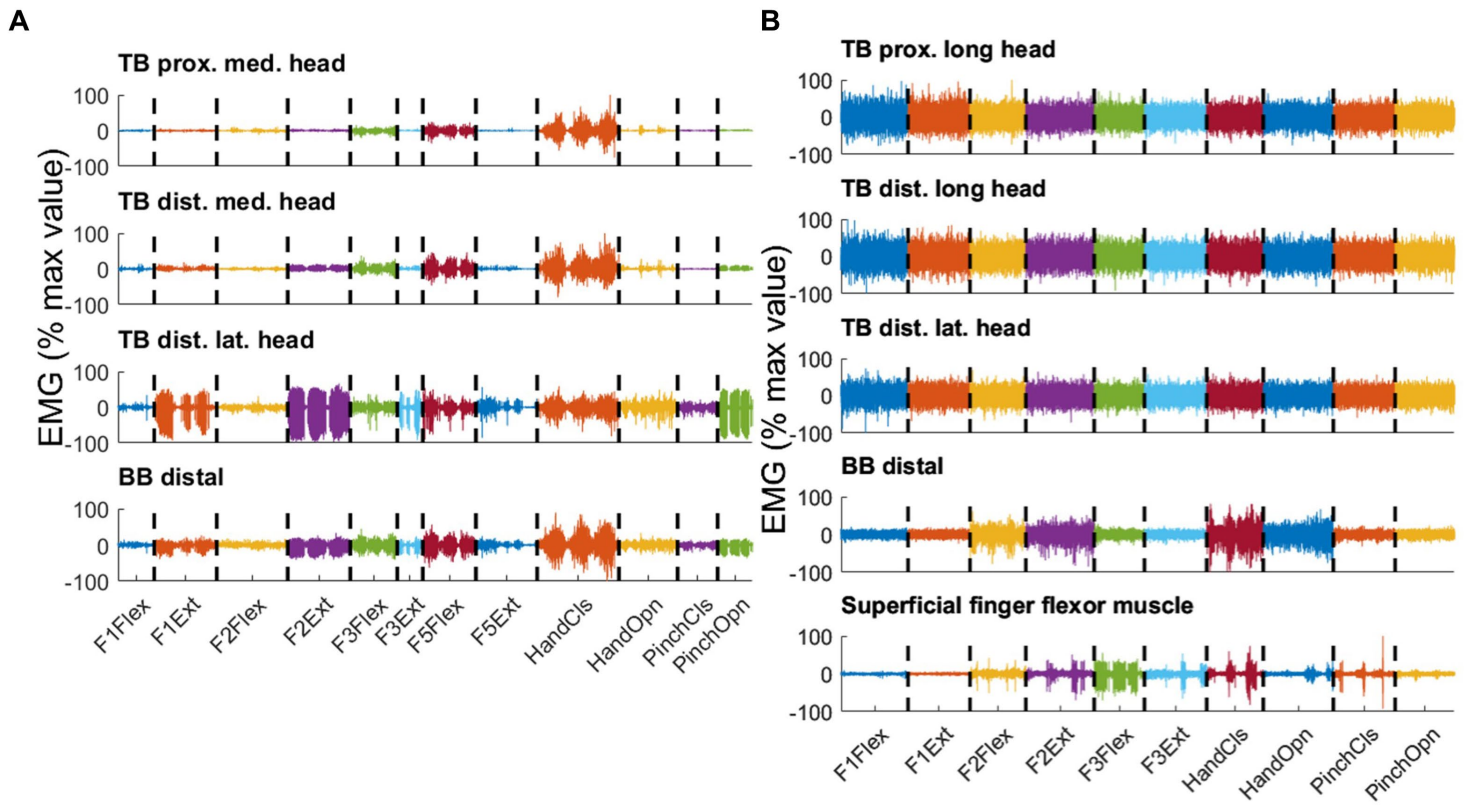
Three consecutive repetitions per type of movement are displayed, distinguished by color for selected pairs of electrodes recorded in parallel on the residual arm of participant P6 **(A)** and the intact arm of a control participant **(B)**. Both PHM-related and IHM-related EMG Signals were concatenated over different types of movements. The different types of movements are flexion (Flex) and extension (Ext) of thumb (F1), index (F2), middle finger (F3) and little finger (F5), closing (CIS) and opening (Opn) of hand and pinch. For each pair of electrodes, the signal is normalized by the maximum peak EMG amplitude found over all movement types and expressed in percentage. **(A)** Upper trace: proximal location on the medial head of triceps brachialis (TB); second trace: distal location on the medial head of TB; third trace: distal location on the lateral head of TB; lowest trace: distal location on BB. **(B)** Upper trace: proximal location on the medial head of biceps brachialis (BB); second trace: distal location on the medial head of BB; third trace: distal location on the lateral head of BB; lowest trace: superficial finger flexor muscle. Note that the lack of phasic activity in the intact arm muscles resulted in high normalized values, misleadingly suggesting high EMG levels.

PHM was associated with reproducible phasic muscle activity that varied with movement type and electrode site. Figure 4A displays typical examples of EMG signals from participant P6 for three consecutive repetitions of various types of PHM, recorded from four pairs of electrodes on the residual upper arm. First, it can be observed that EMG activity is phasic and for a given type of PHM, it is repeatable. These phasic activities are coherent with the kinematics of movement: the EMG bursts show an increasing amplitude at the start of a movement and decreasing amplitude at the end, irrespective of the half-cycle duration (see Figure 2A for illustration). As expected from a muscle directly involved in IHM, similar observations are noted regarding the signals recorded from the forearm superficial finger flexor muscle. Second, for each electrode site, the EMG activity varies according to the type of PHM. Distally on the lateral head of TB (3rd trace), activity was higher for isolated extensions of the thumb and index finger (F1Ext and F2Ext) than for their flexions (F1Flex and F2Flex) but inversely lower when involved in hand opening and closing. Such intra-individual variability has also been observed for the other amputated participants. Finally, as expected from a fusiform muscle such as TB, EMG recordings on a proximal and a more distal site (two top traces for Figure 4A) present similar movement-dependant activity. In Figure 4B, signals were recorded from the intact upper arm muscles and the superficial finger flexor muscle of a control participant executing different types of IHM. As expected from a forearm muscle directly involved in hand movement, the superficial finger flexor muscle exhibited EMG bursts (i.e., phasic activity) for the different types of movements. Interestingly, this activity seemed very similar to the phasic EMG activity measured on the residual upper arm during such movements. In contrast, all sites on TB and BB exhibited mainly tonic activity.

**Figure 5:**
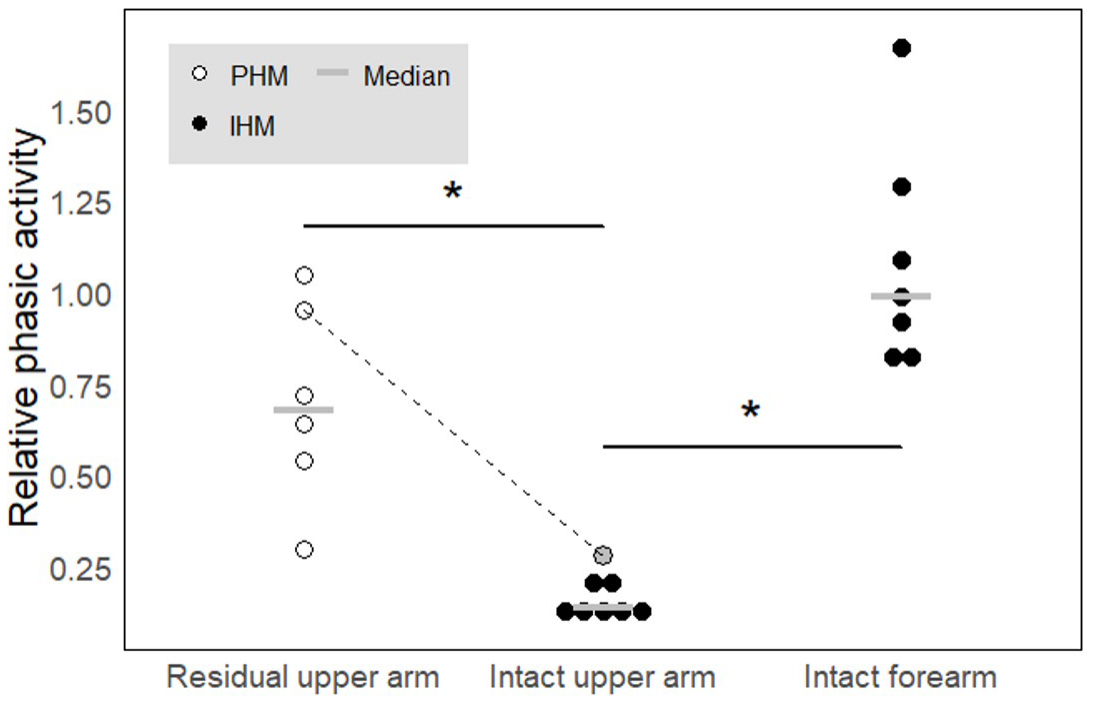
Relative phasic activity across movement types and electrode sites. A high value indicates a phasic profile while a low value indicated a rather tonic profile. In each condition, each circle represents one participant. White circles correspond to amputees while black filled circles correspond to controls. The gray circle added to the intact arm category corresponds to the results obtained for amputee P6 for whom we recorded the residual and intact upper arms in parallel. The dotted line links the data obtained for P6 in the two conditions. The gray horizontal lines represent median values. * indicates significant differences (*p* < 0.05). See text for precise *p*-values.

PHM was associated with relative phasic muscle activity on the residual arm, which was stronger than that associated with IHM in the upper arm and similar to that associated with IHM in the forearm. As could be expected given the EMG traces shown in Figure 4 for individual participants, ‘relative phasic muscle activity’ associated with PHM recorded on the residual limb was found to be significantly higher than that associated with IHM recorded on the intact upper arm (*W* = 42, *p* ≈ 0.003, Table 2^a^; Figure 5). Furthermore, relative phasic muscle activity associated with IHM measured on the intact forearm muscles was significantly greater than that measured in parallel on the intact upper arm (*V* = 28, *p* ≈ 0.02, Table 2^b^). The low values for the intact upper arm muscles are typical of the tonic EMG activity depicted in Figure 2A and Figure 4. We found no significant difference between PHM-related relative phasic muscle activity and IHM-related relative phasic muscle activity measured on the forearm of control participants (*W* = 35, *p* ≈ 0.051, Table 2^c^). Finally, in participant P6 of the amputee group, EMG signals were not only recorded from the residual upper arm but also simultaneously from the intact upper arm during PHM. In Figure 5, results corresponding to P6 are represented by the two circles connected by a line. The difference in relative phasic muscle activity between the residual upper arm during PHM and the intact upper arm during the IHM mimicking the PHM, is consistent with findings from the group comparison.

**Table 2:** Statistical analyses to compare muscle activity patterns during IHM and PHM using the relative phasic activity and its variability across movement-type and electrode-site.

Comparison	Data structure	Type of test	Power
***a***	Non normal	Wilcoxon Rank Sum test (unpaired samples)	*W* = 42
Relative phasic activity residual vs. intact upper arm			*p* ≈ 0.003
			95% CI: [0.19; 0.84]
***b***	Non normal	Wilcoxon	*V* = 28
Relative phasic activity intact forearm vs. intact upper arm		*Signed* Rank test (paired samples)	*p* ≈ 0.02
			95% CI: [0.66; 1.25]
***c***	Non normal	Wilcoxon Rank Sum test (unpaired samples)	*W* = 35
Relative phasic activity intact forearm vs. residual upper arm			*p* ≈ 0.051
			95% CI: [−0.03; 0.75]
***d***	Non normal	Wilcoxon Rank Sum test (unpaired samples)	*W* = 42
Movement-type specificity			*p* ≈ 0.003
			95% CI: [0.13; 0.32]
***e***	Non normal	Wilcoxon Rank Sum test (unpaired samples)	*W* = 39.5
Electrode-site specificity			*p* ≈ 0.01
			95% CI: [0.06; 0.23]

*W, test statistic value for Wilcoxon Rank Sum test; V, test statistic value for Wilcoxon Signed Rank test.*

**Figure 6:**
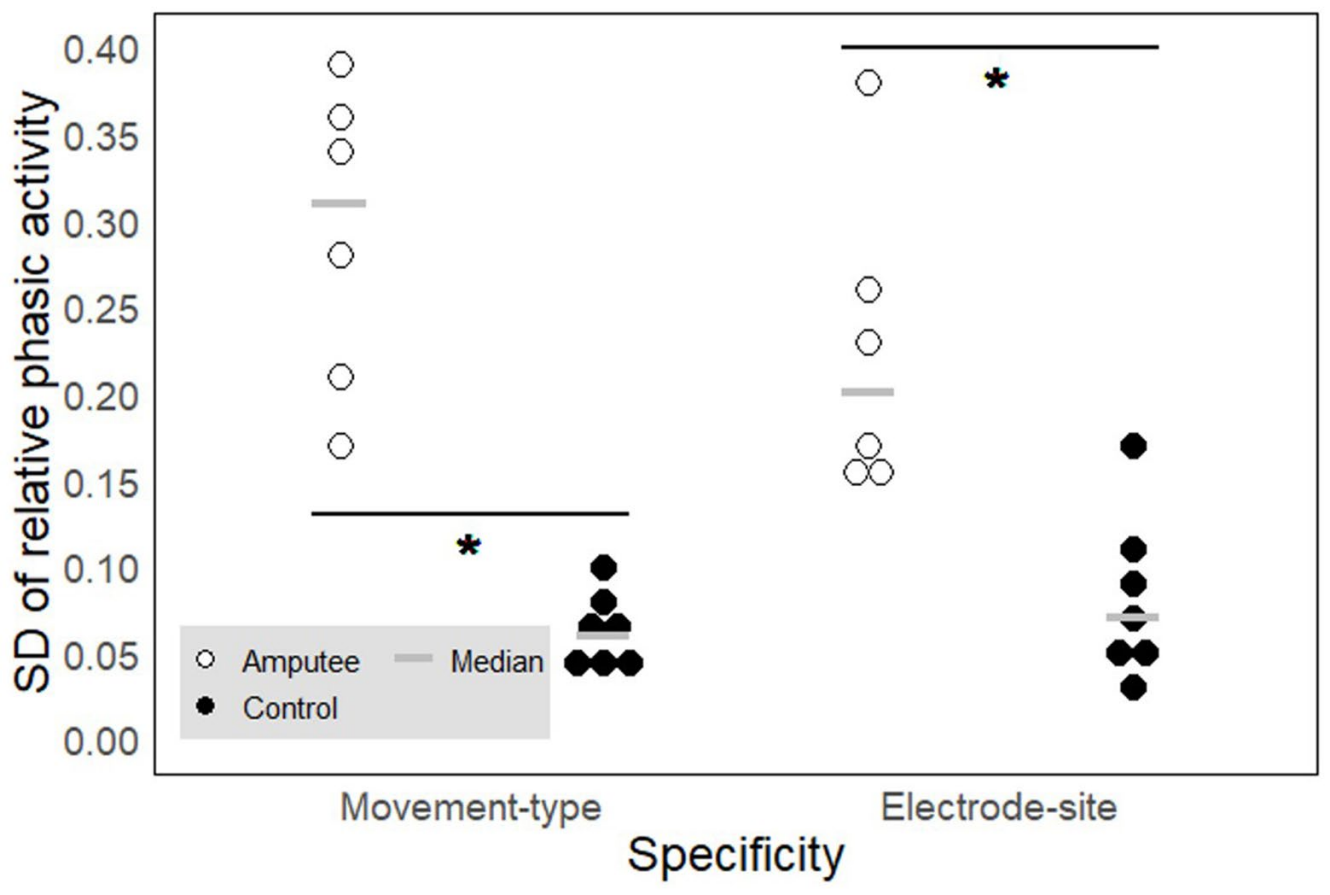
Movement-type and electrode-site specificity of the phasic muscle activity. Each circle represents one participant. The signals were measured on the upper arm for phantom (PHM, amputees, empty circles) and intact (IHM, controls, filled circles) hand movements and expressed in SD of the relative phasic muscle activity. A high SD value reflects a high specificity. The gray horizontal lines represent median values. * indicates significant differences between the two groups (see results section for precise *p*-values).

The relative phasic muscle activity on the upper arm varies more with both the type of movement and the recorded site when associated with PHM than when associated with IHM. We compared movement-type and electrode-site specificities between PHM and IHM in the signals recorded from the upper arm muscles (Figure 6). Both for movement type (*W* = 42, *p* ≈ 0.003, Table 2^d^) and electrode site (*W* = 39.5, *p* ≈ 0.01, Table 2^e^), the results show that EMG patterns associated to PHM and measured on the residual arm were more specific than EMG patterns associated to IHM and measured on the intact upper arm of control participants. This demonstrates that any muscle activity on the residual upper arm during PHM is more directly related to the type of movement and the precise position of the electrode, even within a given muscle, than is the case for the intact upper arm during IHM.

## 4 Discussion

By adopting a neurophysiological perspective in the analysis of activity patterns in the residual upper arm associated with phantom mobility in transhumeral amputees, the first aim of this study was to characterize their nature. For this, we compared the muscle activity patterns on the residual arm during PHM with those during similar IHM on the intact arm of controls. The meta-variables were chosen in order not to base the analyses on comparisons of specific electrode sites between controls and amputees. Instead, we computed the relative phasic muscle activity and its variability across movement types and electrode sites for each participant, that we then compared between groups of participants. This permitted to overcome the unavoidable variability in electrode placements across participants induced by heterogeneity factors such as the anatomy of the upper arm muscles that changes after amputation and as a function of the length of the residual limb.

Our results show significant differences in upper arm muscle activities between IHM and PHM. Muscle activity in the intact upper arm during IHM was characterized by highly tonic signals and uniform patterns depicted through low movement-type and low electrode-site specificities, which could be expected since upper arm muscles do not control hand movements in intact limbs. On the other hand, during PHM, the residual upper arm muscle activity appeared to be phasic and more precisely highly correlated with the kinematics of the movement, irrespective of the duration of the movement half-cycle. When quantified, this PHM-related activity exhibits significantly higher relative phasic activity as well as both movement-type and electrode-site specificities. Moreover, the relative phasic activity values were comparable with those seen in the forearm and intrinsic hand muscles during IHM. In the intact upper arm, while the low electrode-site specificity can be explained by the wide distribution of the muscle fibers of each individual motor unit throughout the entire muscle, allowing for homogeneous muscle contraction, the low movement-type specificity is consistent with the expectation that separate movements of intact adjacent fingers are associated with tonic activity patterns in the upper arm, allowing to stabilize the more proximal joints. If the activity in the residual upper arm muscles during PHM had remained with the same tonic synergistic activity as the one before amputation during IHM, one should have found similar results. However, as this is not the case, our results do not support residual synergistic activity as being the sole reason for the existence of phasic muscle activities and localized muscle contractions in the residual upper arm during PHM, but rather suggest neural reorganization.

Neural reorganization after amputation is a topic of ongoing debate. Reorganization at the cortical level has been supported by numerous reports where the deafferented area in M1 and S1 would be invaded by the neighboring areas (i.e., residual limb and face) erasing the functional representation of the lost limb (Ojemann and Silbergeld, 1995; Lotze et al., 2001; Navarro et al., 2007). These intraoperative studies of cortical mapping were completed by more recent studies using transcranial magnetic stimulation (TMS). Applying TMS over the area of M1 that controlled the hand before amputation, resulted in muscle contractions in the residual upper arm (Gagné et al., 2011) while evoking a sensation of movement in the phantom hand (Mercier et al., 2006). However, Makin’s team recently showed that the apparent large-scale reorganization observed at the cortical level might in fact be the result of an artifact (Makin and Krakauer, 2023; Schone et al., 2023). Thus, the previous TMS results, as well as the observed muscle contractions on the residual limb associated with PHM might be the results of reorganization at other levels, in particular at the periphery. Moreover, exclusive central reorganization cannot explain the high electrode-site specificity of the patterns observed in our study. Instead, the present findings strongly suggest that specific sub-volumes within upper arm muscles are selectively activated during PHM. This hypothesis finds support in research on both human and non-human primates. First, during phantom hand movements, activity in the severed median and ulnar nerves has been measured in human amputees (Dhillon et al., 2004; Jia et al., 2007). Second, surgical muscle reinnervation targeting specific nerve fascicles enables independent control of focal muscle compartments (Bergmeister et al., 2017; Woo et al., 2016). Finally, natural peripheral neuromuscular reorganization, observed in monkeys after transhumeral amputation, involved axonal sprouting of severed motoneurons that controlled hand muscles before amputation and recapture of surrounding denervated muscle fibers in the residual upper arm, thereby forming new neuromuscular junctions (Qi et al., 2004; Wu and Kaas, 2000). It is worth noting that due to relatively distal localization of motor endplates in the upper arm muscles (1/3 of the upper arm length above the elbow joint, Guzmán et al., 2011), many muscle fibers are likely left without innervation after transhumeral amputation, increasing the likelihood of their capturing by axotomized spinal motoneurons. Consequently, cortical motoneurons initially controlling hand muscles would eventually govern muscle volumes in the residual limb, which could explain the similarity in phasic muscle activities between the residual upper arm during PHM and intact forearm during IHM. While the connection between natural sprouting- and recapture-mediated peripheral reorganization and phantom mobility has not been explicitly established, the present study, and precisely the specific and focal activity patterns observed in the residual upper arm muscles during phantom limb movement execution, aligns with such reorganization. However, further research is required to confirm this hypothesis.

Our study may contain potentially limiting methodological issues that should be discussed.

### 4.1 Mimicking with the intact hand

This method is often used in the literature since it is the only way to objectively determine the timing of phase changes in order to cut the EMG activity into half cycles. Yet, one might raise the question whether synchronous mimicking could have influenced the EMG patterns on the residual limb. This seems not to be the case, as shown, for instance, by Jarrassé et al. (2017b) in their EMG-pattern classification study. In order to correctly identify phantom movement types, they used mimicking during the *learning phase* of the classifier but not during testing, and the high success rate of the classifier in correctly recognizing the executed movement types showed that mimicking with the intact hand did not influence the EMG patterns associated with phantom hand movements.

### 4.2 Suitability of the electrode sites

Electrode placement can affect the content and quality of EMG signals. It is precisely for this reason that we used an ‘unusual’ electrode placement on the intact arm to record adjacent volumes on the same muscle, resembling the electrode sites on the residual arm of amputees. Yet, we did not observe identical results, particularly with regard to the electrode specificity of the EMG patterns. The poorly specific EMG profiles found on the intact arm were expected in the biceps and triceps muscles due to their tonic type activities during finger and hand movements. Despite the similar placements of electrodes on the residual arm, EMG variability across recording sites is much higher. One might still raise the question whether we may have missed information, in particular because we did not cover the entire surface of the residual limb. Yet, we used 8–15 pairs of electrodes (depending on the length of the residual limb), carefully placed using palpation. Jarrassé et al. (2018), using the same method, showed that this was sufficient to classify 8 different phantom movements with a success rate of over 85%. Therefore, we believe that in the present study, most relevant information was captured.

### 4.3 Choice of the control condition

In the introduction section, we mentioned the potential risks associated with selecting the contralateral arm of amputated participants as the control condition. In order to avoid biases in our results, comparison between PHM and an intact residual limb movement involving the biceps and triceps would have been optimal. However, residual limb movements concern the shoulder articulation (ante/retropulsion and adduction) for which the deltoid muscle alone usually is sufficient, especially after transhumeral amputation. Finally, while comparing muscle activity patterns during PHM to those during phantom elbow movements may seem plausible, it is also not ideal for two reasons. First, phantom segments and joints as well as their mobility are mostly present at the most distal segments of the upper limb (Touillet et al., 2018, study on 76 amputees). Thus, most amputees cannot perform phantom elbow movements for the simple reason that they either do not feel the phantom elbow or cannot move it. Among our six participants, only two perceived their phantom elbow (P4 and P5) and only 1 of them could execute elbow flexions and extensions (P5). When an amputee is asked to perform a phantom movement at a joint that they do not feel or cannot move, either there is no EMG signal (Reilly et al., 2006) or this leads to major coactivation of the residual limb muscles as the participant attempts to perform this impossible task. Second, even when the phantom elbow is present and can be moved, the residual limb muscles acting on the elbow joint are not activated in the same way as before amputation, as transhumeral amputation severed these muscles and expectedly removed many of their motor endplates. Therefore, capturing of denervated muscle fibers by severed spinal motoneuron sprouting (whether originally projecting on hand, forearm or the same upper arm muscles) can be expected. All these reasons make the use of phantom elbow flexions as a control condition very doubtful. We therefore believe IHM of control participants to be the most reasonable control condition for comparing patterns of muscle activity on upper arms.

### 4.4 Limited number of participants

The limited sample size in terms of participants in research focusing on specific populations is a well-known problem, in particular the recruitment of participants satisfying our inclusion criteria and willing to give half a day of their time. Although a larger population would have been preferable, other articles concerned similar number of participants (Gagné et al., 2009; Jarrassé et al., 2018, 2017a; Reilly et al., 2006; Rossel et al., 2023; Touillet et al., 2018). If we had not found a significant result in the present study, this limited number of participants would have been a matter of great concern. However, despite differences across participants in delay since amputation, level of upper arm amputation, pain treatment, and age, all six participants showed much greater EMG variability across electrode localisations and movement types during phantom hand movement execution compared to intact hand movements in non-amputated control participants. Concerning pain treatment, although this might reduce the intensity of phantom sensations (or even sensations in general), it seems not to influence the capacity of producing phantom movements, as shown in the epidemiological study of Touillet et al. (2018). Finally, regarding the influence of age on neuromuscular mechanisms, two of our participants were older than 70 years which means that they might have been affected by the age-related phenomenon of denervation/reinnervation. This phenomenon involves muscle fibers that were once part of fast motor units being reinnervated by the motoneuron of a slow motor unit, eventually leading to an increase in the size of the latter (Piasecki et al., 2016). This mechanism ultimately increases the mean size of motor units, which explains the decline in motor control precision with age. However, we observed the opposite in the present study: contrary to intact upper arm muscles, smaller muscular volumes contract differently for different types of phantom hand movements, which strongly suggests a reduction in the size of motor units. We therefore believe that the observed phenomenon in the present study is as robust as those previously published on phantom mobility.

### 4.5 Limited number of trials

The limited sample size (10 repetitions per type of movement) was a necessity. Indeed, untrained phantom movements are slow (performing a cycle of finger flexion/extension or hand opening/closing takes on average 6 s and can sometimes take more than 30 s). They are reported to be exhausting (De Graaf et al., 2016), and the required 10 repetitions are sometimes impossible to perform consecutively because they are too strenuous. On the other hand, in the absence of signs of fatigue, EMG patterns show remarkable reproducibility across movement cycles even when not performed consecutively and 10 repetitions are sufficient to allow classification of these patterns and thus define the movement performed as distinct from the others (Jarrassé et al., 2017a,b). To maintain the reproducibility of the EMG patterns and thus limit the effects of fatigue on the recorded EMG patterns, if we observed any signs of visible fatigue such as slower movements, slight tremors or freezing (i.e., the hand feels as clamped in a vice and cannot be moved), we halted the recording and provided the participants with a short break. To limit fatigue even further after the break, we suggested that participants perform another type of phantom movement instead of directly resuming the original 10-cycle series. Due to the necessary precautions taken, the recording of all potential phantom movements required at least 1.5 h for each participant. Requesting additional recordings seemed likely to induce fatigue.

### 4.6 Randomization of trials

Our experimental design would be insufficient in a study on intact participants, but in the case of phantom movements in untrained amputees, similar randomization is more difficult to implement. Indeed, our participants needed intense concentration because none of them were used to producing phantom movements. Thus, if we had to randomize our 10 repetitions without increasing the number of trials, we would need additional periods of concentration between each movement execution, which would inevitably lengthen an already long experimental session and potentially induce fatigue that we were in fact trying to avoid. Jarrassé et al. (2017a,b) used EMG activity associated with phantom hand movements in transhumeral amputees and to test whether the observed muscle activity patterns on the residual upper arm were classifiable (and thus reproduceable) as a function of the executed type of movement. For this, during the learning phase of the classifier, they used either 2 cycles or 10 cycles executed in a row, and then tested the classifier with movements performed *one at a time* (i.e., without repetitions). The success rate of classification was about 80% when considering six different types of movement, which was similar to general classification rates in the literature of surface EMG activity in forearm amputees who still have residual hand muscles (Cipriani et al., 2011; Smith and Hargrove, 2013). This shows that the patterns of EMG activities are similar and reproducible whether the phantom movements are executed in a row or randomly, one at a time. Thus, we believe that randomization would not have fundamentally changed the outcome of this study.

### 4.7 Use of prosthetic limbs

Some of our participants were using a prosthetic hand daily and it might be argued that the specificity of EMG associated with phantom hand and finger movements is correlated to the ability of the amputee to use a myoelectric hand. But phantom mobility and the control of current conventional myoelectric prostheses do not influence each other (Touillet et al., 2018). This is particularly the case of transhumeral amputees. Indeed, the control of their prosthetic hand and wrist movements is based on the production of muscle activity in the residual upper arm muscles for which the recorded EMG signal should reach a certain threshold. The signals are typically recorded by only two pairs of electrodes placed on antagonistic muscles, i.e., the biceps and triceps in transhumeral amputees. Even for the amputees who use a complex poly-digital hand prosthesis (e.g., P5), the different finger movement configurations are controlled through simple succession of basic co-contractions of the two antagonistic muscles. This control does not allow the use of the poly-digital hand prosthesis to its full potential. On the other hand, the patterns we recorded during PHM through the use of multiple electrodes are sufficiently complex to be classified as distinct patterns that are systematically associated with *specific individualized finger movements* (Jarrassé et al., 2017a,b). Therefore, in no case can the distributed muscle activity we observed be a learned feature associated with the use of their myoelectric prosthesis. Moreover, transhumeral amputees can clearly distinguish between the action required to elicit prosthetic movements and the action that provoked the natural somatosensory feedback involved in voluntary movement of the phantom hand (Touillet et al., 2018). In the former, participants concentrate on producing a specific EMG intensity set by the system’s threshold, which is challenging and must be learned (de Graaf et al., 2004). It is the visual feedback of the prosthesis moving or the auditory feedback (the beep) of the system switching configuration that validates the action. In the latter case, the EMG pattern appears naturally inducing somatosensory feedback from the tissues in movement in the residual arm, thereby giving feedback on the generated movement without the need for a learning phase (De Graaf et al., 2016). Phantom mobility can still be trained (as an intact movement can be), but even then Rossel et al. (2023) showed that daily training for 2 months did not fundamentally alter EMG patterns, although it did increase PHM velocity. All these findings highlight the primary advantages of PHM-based myoelectric prosthesis control over conventional methods, particularly for individuals with transhumeral amputations.

In conclusion, the present study strongly suggests that the muscle activity systematically associated with phantom hand mobility could, at least partly, originate from the sprouting of axotomized spinal motor neurons and the retargeting of residual muscle fibers. To advance prosthesis control, rehabilitation protocols should incorporate training for phantom mobility which increases the number of executable phantom movement types (Touillet et al., 2018) and consequently enhance the capacity of control over the phantom hand. This approach holds promise for mitigating residual muscle atrophy and facilitating the rapid emergence of muscle sub-volumes by stimulating potential axonal sprouting and reinnervation of residual muscle fibers by severed motoneurons. Given that peripheral neural reorganization has been found to occur during a so-called “early” period, which may persist for up to 2 years following complete axotomy (Delmotte et al., 2009), addressing phantom limb mobility should start promptly after amputation.

## Data Availability

The raw data supporting the conclusions of this article will be made available by the authors, without undue reservation.

## Footnotes

^1^ https://www.cyberglovesystems.com/cyberglove-ii
